# QTc and psychopharmacs: are there any differences between monotherapy and polytherapy

**DOI:** 10.1186/1744-859X-6-13

**Published:** 2007-05-03

**Authors:** Jadranka Čulav Sumić, Vesna Barić, Petar Bilić, Miroslav Herceg, Mirna Sisek-Šprem, Vlado Jukić

**Affiliations:** 1 Vrapče Psychiatric Hospital, Bolnička 32, 10090 Zagreb, Croatia

## Abstract

**Background:**

Some psychotropic drugs are connected with prolongation of QT interval, increased risk of cardiac arrhythmias and greater incidence of sudden death, especially when used in combination. Concomitant use of antipsychotics and antidepressants is not rare in our clinical practice. The study compares the length of QT interval in patients on monotherapy with an antipsychotic or an antidepressant and patients taking polytherapy (an antipsychotic agent combined with an antidepressant).

**Methods:**

Sixty-one hospitalized women who met the ICD-10 criteria for schizophrenia, schizoaffective psychosis, delusional disorder and mood disorder were included in the study. The monotherapy group was consisted of thirty-two women treated with an antipsychotic or an antidepressant while the polytherapy group was composed of twenty-nine women treated with an antipsychotic agent plus an antidepressant. Two electrocardiograms (ECGs) were obtained for each patient: the first was carried out before the treatment and the second after two weeks of treatment.

Statistical analysis was carried out by SPSS program and included unpaired and paired t test and Fisher's exact test.

**Results:**

Mean baseline QTc values did not differ between the groups (439 ± 22 ms was the same value found in the both groups; unpaired t test, p > 0.5). Mean QTc intervals after two weeks of treatment were also similar (439 ± 24 ms in the monotherapy group and 440 ± 20 ms in the polytherapy group; unpaired t test, p > 0.5). Fisher's exact test did not reveal significant difference in the number of patients with borderline (451–470 ms) or prolonged (> 470 ms) QTc between groups, neither before treatment nor after two weeks of treatment. Twenty two women of the total of sixty one patients (36%) had QTc > 450 ms before applying therapy.

**Conclusion:**

We did not find significant QT prolongation in our patients after two weeks of treatment with antipsychotics and/or antidepressants. The QTc interval length did not differ significantly in the monotherapy and the polytherapy group. More than one third of included women exceeded the threshold value of borderline QTc interval (450 ms) before starting treatment. This finding calls for caution when prescribing drugs to female psychiatric patients, especially if they have other health problems.

## Background

Psychotropic drugs are among medications connected with prolongation of the QT interval and greater occurrence of sudden cardiac death [1-7]. The QT interval is the sequence of the ECG from the beginning of the QRS complex to the end of the T wave and represents the temporal equivalent of ventricular depolarization and repolarization. Its value corrected for heart rate is referred as corrected QT interval (QTc). There is no consensus about the upper physiological limit for QTc [8]. European Medicines Agency quotes different possible upper values (450 ms, 480 ms, and 500 ms) and calls for caution when change from baseline exceeds 30–60 ms [9]. Significant QT prolongation ("long QT syndrome", LQTS), inherited or acquired, is associated with the increased susceptibility to ventricular tachyarrhythmia "torsade de pointes" (TdP) that either resolve spontaneously or deteriorate into ventricular fibrillation and sudden death. In comparison to men, women are at higher risk for developing TdP because the feminine gender is associated with a longer baseline QT interval, perhaps due to differences in circulating sex hormones [10-13]. For females, QTc interval values more than 450 ms are commonly used as borderline and those over 470 ms as prolonged [14-16].

Congenital forms of LQT syndrome are due to autosomal recessive (Jervell and Lange-Nielsen syndrome) or autosomal dominant (Romano-Ward syndrome) mutations of several genes encoding for cardiac ion channels with consequent disturbances in electrical activity of the heart [17-22]. LQTS mutation carriers are present in one of 1000 to 3000 individuals [23].

Acquired long QT syndrome occurs when one or more risk factors, such as drugs that block certain cardiac ion channels, provoke a prolonged QT interval. Common causes of acquired LQTS are several classes of drugs, e.g. Class I and III antiarrhythmics, macrolides antibiotics, antihistamines, antipsychotics and antidepressants [24]. International Registry for Drug-Induced Arrhythmias by the University of Arizona [25] put some antipsychotics among the drugs with the most prominent arrhythmogenic activity (haloperidol, chlorpromazine, pimozide and thioridazine); less capable to induce arrhythmias are clozapine, lithium, quetiapine, risperidone, venlafaxine and ziprasidone. Antidepressants (amitriptyline, clomipramine, citalopram, fluoxetine, paroxetine and sertraline) are at lower risk if they are not combined with other risk factors known to prolong the QT interval (e.g. concomitant therapy with QTc prolonging drugs or inhibitors of cytochrome 450 enzymes, bradycardia, presence of congenital LQTS, and electrolyte imbalance like hypokalaemia and hypocalcaemia). Some studies pointed out the greater possibility for cardiac arrhythmias when antipsychotic drugs are combined with antidepressants [26].

Because concomitant use of antipsychotics and antidepressants are not infrequent in our clinical practice we decided to explore are there any differences in the length of QTc between patients on monotherapy with an antipsychotic or an antidepressant and patients treated with combination of these drugs (an antipsychotic plus an antidepressant).

## Methods

A prospective investigation was performed in Psychiatric hospital Vrapče, Zagreb. Sixty one patients, all women, were included in the study, as consecutively received patients from January to September 2006. Informed consents were obtained and the local ethic committee approved the investigation. The patients met the ICD-10 (International Classification of Disease, 10^th ^revision) criteria for schizophrenia, schizoaffective psychosis, delusional disorder and mood disorder. According to patient's history, clinical examination and laboratory tests, patients with liver or renal disorders, cardiovascular disease or psychoactive drugs dependence were not included in the study. The use of depot-therapy in the month prior to investigation and the use of fluoxetine (because of its long half-life) were the exclusion criteria also.

The patients were free of drugs minimum 48 hours before the first ECG and the blood samples were taken. Only the use of lorazepam (up to 7.5 mg/d) was permitted. The second ECG was carried out after two weeks of treatment. The group 1 was on monotherapy (treated with an antipsychotic or an antidepressant). The group 2 was on polytherapy (treated with an antipsychotic and an antidepressant). As concomitant therapy in both groups the use of biperiden or lorazepam was possible if necessary. All daily antipsychotic and antidepressant doses were converted to defined daily dose equivalents (DDD), as defined by the World Health Organization, and the current daily dose was categorized into less than one DDD equivalent and one or more DDD equivalents [27].

All patients had normal liver and renal functions according to normal values of transaminases, blood urea nitrogen and creatinine. The serum levels of potassium, sodium and calcium ions were determined. Body weight and height were measured and body mass index (BMI) was calculated. ECG was performed by routine clinically used 12-lead electrocardiogram apparatus which automatically calculates the QTc interval. We are aware that the method used is a limitation of this study because the measurement by the cardiologist could have been more accurate [28]. The length of QT interval was compared before and after treatment in the same group and between the groups; differences were statistically analysed. The statistical analysis was done using SPSS program 12.0 and included independent and dependent t test and Fisher's exact test.

## Results and discussion

### Characteristics of patients and applied therapy

Sixty one patients were included in the study. Thirty two women were receiving an antipsychotic or an antidepressant (group 1) and twenty nine women were treated with an antipsychotic in combination with an antidepressant. The two groups did not differ significantly with respect to age, duration of illness, BMI, smoking status and doses of psychopharmacs converted to DDD equivalents (Table 1). In twenty patients (33%) doses of applied psychotropic drugs were above DDD while forty one patients (67%) had equal or smaller doses in comparison to DDD. Table 2 show the frequency of applied antipsychotics and antidepressants respectively in the both groups.

**Table 1:** Characteristics of patients

	*Group 1 (N = 32)*	*Group 2 (N = 29)*	
*Characteristic*	*Patients in monotherapy*	*Patients in polytherapy*	*p
Age, range (yr)	27–69	27–70	
Age, mean ± SD (yr)	48.3 ± 8.8	48.6 ± 11.3	0.771
Duration of illness, mean ± SD (yr)	10.8 ± 7.3	10.7 ± 9.1	0.950
BMI, mean ± SD	25.1 ± 5.5	27.9 ± 5.5	0.059
			**p
			
Smoking present, N (%)	16 (50.0)	15 (51.7)	1.000
Applied dose > DDD:			
Dose of AP > DDD, N (%)	7 (21.9)	8 (27.6)	0.767
Dose of AD > DDD, N (%)	9 (28.1)	11 (37.9)	0.586

*p – 2-tailed t test; **p – 2-tailed Fisher Exact test; BMI – "body mass index";

DDD – "defined daily dose"; AP – antipsychotic; AD – antidepressant

**Table 2:** Characteristics of applied therapy

	*Group 1 (N = 32)*		*Group 2 (N = 29)*		
*Psychotropic drug*	*Patients in monotherapy*		*Patients in polytherapy*		
	
	*N of patients*	*dose range (mg/d)*	*N of patients*	*dose range(mg/d)*	
					*AD used*
					
*Antipsychotic*					
Ziprasidone	3	120–160	1	120	map
Olanzapine	4	10–20	7	5–15	Mir,ven,par,fluo
Clozapine	1	100	-	-	-
Risperidone	3	3–5	1	4	map
Sulpiride	1	400	3	50–200	ser,clo
fluophenazine	7	5–8	6	2–15	tia,ser,par,map
Haloperidol	3	6–15	4	4–15	mir,map,esc,clo
Promazine	-	-	4	75–350	tia,ven,map
Quetiapine	-	-	2	300–500	par,fluo
zuclopenthixol	-	-	1	10	Fluo
					
					*AP used*
					
*Antidepressant*					
Mirtazapine	2	30	3	15–30	ol,hal
Fluvoxamine	1	150	-	-	-
Tianeptin	2	37.5	2	37.5	flu,pro,
Sertraline	2	50–100	2	50	sul,flu
Venlafaxine	2	37.5–75	4	37.5–150	ol,pro
Paroxetine	-	-	4	10–40	Ol,flu,que
Maprotiline	1	100	7	50–100	zip,ris,flu,hal
Fluoxetine	-	-	3	20–40	ol,que,zuc
escitalopram	-	-	1	15	Hal
clomipramine	-	-	3	25	sul,hal

AD – antidepressant: map-maprotiline, mir-mirtazapine, ven-venlafaxine, par-paroxetine, fluo-fluoxetine, ser-sertraline, clo-clomipramine, tia-tianeptin, esc-escitalopram

AP -antipsychotic: ol-olanzapine, hal-haloperidol, flu-fluophenazine, pro-promazine, sul-sulpiride, que-quetiapine, zip-ziprasidone, ris-risperidone, zuc-zuclopenthixol

### QTc interval

Mean baseline values of QTc in group 1 (439 ± 22 ms) and group 2 (439 ± 22 ms) were similar (independent t test p = 0,953) (Table 3). There were no significant differences in the length of QTc between the groups after two weeks of treatment also: the mean values were 439 ± 24 ms in the group 1 and 440 ± 20 ms in the group 2 (independent t test p = 0,878 (Table 3). In group 1 the length of QTc before and after treatment was similar (dependent t test p = 0.989); the same was observed in group 2 (dependent t test p = 0.812). The two groups did not differ significantly in the number of patients with QTc > 470 ms, not before therapy (Fisher's exact test p = 0.600) neither after two weeks of treatment (Fisher's exact test p = 0.674). There were three women (9.4%) in the group 1 with the QTc prolongation more than 30 ms from the baseline value (prolongations were 30, 32, and 87 ms) and the same number was found in the group 2 (10.3%), (prolongations were 44, 66, and 66 ms). Mean values of QTc prolongation in the group 1 and group 2 were 8 ± 17 ms and 9 ± 19 ms respectively (independent t test p = 0.840).

**Table 3:** Characteristics of QTc interval

	*Group 1 (N = 32)*	*Group 2 (N = 29)*	
*Characteristic*	*Patients in monotherapy*	*Patients in polytherapy*	*p
QTc I, mean ± SD (ms)	439 ± 22	439 ± 22	0.953
QTc II, mean ± SD (ms)	439 ± 24	440 ± 20	0.878
***p	0.989	0.812	
			
QTc prolongation, mean ± SD (ms)	8 ± 17	9 ± 19	0.840
			
			**p
			
QTc I > 450 ms, N (%)	12 (37.5)	10 (34.5)	1.000
QTc II > 450 ms, N (%)	10 (31.3)	9 (31.0)	1.000
QTc I 451–470 ms, N (%)	11 (34.4)	8 (27.6)	0.593
QTc II 451–470 ms, N (%)	6 (18.7)	7 (24.1)	0.757
QTc I > 470 ms, N (%)	1 (3.1)	2 (6.9)	0.600
QTc II > 470 ms, N (%)	4 (12.5)	2 (6.9)	0.674
QTc II – QTc I > 30 ms, N (%)	3 (9.4)	3 (10.3)	1.000

*p – 2-tailed unpaired t test; **p – 2-tailed Fisher Exact test; ***p – 2-tailed paired t test; QTc I – baseline QTc; QTc II – QTc after two weeks of treatment

Our study did not reveal significant differences in the mean QTc length between women treated with antipsychotics or antidepressants and women who were treated with both of these drugs. There was no significant QT prolongation after two weeks of treatment in the both groups too. No one patient had QTc = 500 ms. Eight patients of sixty one patients included in the study (13%) had QTc intervals > 470 ms and/or the QTc prolongation of 30 ms or more from the baseline value. Five of these eight patients were from the monotherapy group: three women who were taking fluphenazine (7.5 mg/d), venlafaxine (37.5 mg/d) or mirtazapine (30 mg/d) had hypocalcaemia, one woman was on ziprasidone (160 mg/d) and the last one (on fluphenazine 7.5 mg/d) had borderline QTc before starting treatment. The rest three patients were in the polytherapy group: one woman was treated with promazine (200 mg/d) and maprotiline (100 mg/d) and had positive family history of sudden father's death; one patient was on high antidepressant therapy: paroxetine (40 mg/d) in combination with mirtazapine (30 mg/d) and olanzapine (5 mg/d); the third one was treated with promazine (75 mg/d) and venlafaxine (75 mg/d). All of these eight patients had normal potassium and sodium serum levels.

One potential explanation why we did not observe significant QT prolongation in women on combined psychotropic therapy could be the dose of psychopharmacs applied. In two third of included patients doses of antipsychotics and antidepressants were equal or below DDD, in the group 1 and 2. The quantity of drug given to patient was determined by psychiatrist who cured the patient and was clinically determined. Further more, some authors point out that DDD equivalents are smaller than chlorpromazine or haloperidol equivalents used in some previous studies [29]. The other explanation could be the relatively small number of encompassed patients.

Hennessy and al. [4] found that treated schizophrenic patients have longer QTc intervals and higher rates of cardiac arrhythmias than control subjects but they could not determine whether that finding was connected with schizophrenia or its treatment. We found in our study that a great proportion of included patients (more than one third) exceeded the threshold of borderline QTc values (> 450 ms) prior to treatment, and the mean duration of psychiatric illness was more than 10 years. Possible explanation for this finding could be that patients with schizophrenia are at higher risk for other illnesses (e.g. atherosclerosis and cardiac abnormalities) than people in the general population [30,31].

## Conclusion

We did not find significant differences in QTc length after two weeks of treatment between patients treated with antipsychotics or antidepressants and those treated with combinations of these drugs. No one patient had QTc interval equal or longer than 500 ms, not before therapy neither after two weeks of therapy, but more than one third of included women had borderline QTc values before starting therapy. Our results encourage us in our clinical work but not in manner to be less cautious when prescribing psychopharmacs, especially in patients with renal, hepatic, cardiovascular or other health problems.

## Competing interests

The author(s) declare that they have no competing interests.

## Authors' contributions

JČS conceived of the study, performed the statistical analysis and helped to draft the manuscript. VB, PB, MH and MSŠ participated in the design of the study and collecting patients for inclusion and helped to draft the manuscript. VJ participated in the design of the study and interpretation of data. All authors read and approved the final manuscript.

## References

[B1] Straus SMJM, Sturkenboom MCJM, Bleumink GS, Dieleman JP, van der Lei J, de Graeff PA, Kingma JH, Stricker BHCh (2005). Non-cardiac QTc-prolonging drugs and the risk of sudden cardiac death. European HeartJournal.

[B2] Straus SMJM, Bleumink GS, Dieleman JP, van der Lei J, 't Jong GE, Kingma JH, Sturkenboom MCJM, Stricker BHCh (2004). Antipsychotic and the risk of sudden cardiac death. Arch Intern Med.

[B3] Gardner DM, Baldessarini RJ, Waraich P (2005). Modern antipsychotic drugs: A critical overview. CMAJ.

[B4] Hennessy S, Bilker WB, Knauss JS, Margolis DJ, Kimmel SE, Reynolds RF, Glasser DB, Morrison MF, Strom BL (2002). Cardiac arrest and ventricular arrhythmia in patients taking antipsychotic drugs: cohort study using administrative data. BMJ.

[B5] Fayek M, Kingsbury SJ, Zada J, Simpson GM (2001). Cardiac effects of antipsychotic medications. Psychiatr Serv.

[B6] Vieweg WVR, Wood MA (2004). Tricyclic antidepressants, QT interval prolongation and torsade de pointes. Psychosomatics.

[B7] Glassman AH, Bigger JT (2001). Antipsychotic drugs: prolonged QTc interval, torsade de pointes, and sudden death. Am J Psychiatry.

[B8] Drew BJ, Califf RM, Funk M, Kaufman ES, Krucoff MV, Laks MM, Macfarlane PV, Sommargren C, Swiryn S, Van Hare GF (2004). Practice standards for electrocardiographic monitoring in hospital settings. Circulation.

[B9] Committee for Medicinal Products for Human Use (2004). The Clinical Evaluation of QT/QTc Interval Prolongation and Proarrhythmic Potential for Non-Antiarrhythmic Drugs. http://www.emea.eu.int/pdfs/human/ich/000204en.pdf..

[B10] Cheng J (2006). Evidences of the gender-related differences in cardiac repolarization and the underlying mechanisms in different animal species and human. Fundam Clin Pharmacol.

[B11] Pham TV, Rosen MR (2002). Sex, hormones, and repolarization. Cardiovasc Res.

[B12] Drici M-D (2001). Influence of gender on drug-acquired long QT syndrome. Eur Heart J Supplements.

[B13] Ito H, Kono T, Ishida S, Maeda H (2004). Gender difference in QTc prolongation of people with mental disorders. Ann Gen Hosp Psychiatry.

[B14] Van Mieghem C, Sabbe M, Knockaert D (2004). The clinical value of the ECG in noncardiac conditions. CHEST.

[B15] Llerena A, Berecz R, Dorado P, De la Rubia A (2004). QTc interval, CYP2D6 and CYP2C9 genotypes and risperidone plasma concentrations. J Psychopharmacol.

[B16] O'Brien P, Oyebode F (2003). Psychotropic medication and the heart. Adv Psychiatr Treat.

[B17] Moss AJ (2003). Long QT syndrome. JAMA.

[B18] Kass RS, Moss AJ (2003). Long QT syndrome: novel insights into the mechanisms of cardiac arrhythmias. J Clin Invest.

[B19] Moss AJ, Kass RS (2005). Long QT syndrome: from channels to cardiac arrhythmias. J Clin Invest.

[B20] Clancy cE, Kass RS (2005). Inherited and acquired vulnerability to ventricular arrhythmias: cardiac Na+ and K+ channels. Physiol Rev.

[B21] Priori SG (2004). Inherited Arrhythmogenic Disease: The complexity beyond monogenic disorders (reviews). Circ Res.

[B22] Roden DM, Viswanathan PC (2005). Genetics of acquired long QT syndrome. J Clin Invest.

[B23] Yang P, Kanki H, Drolet B, Yang T, Wei J, Viswanathan PC, Hohnloser SH, Shimizu W, Schwartz PJ, Stanton M, Murray KT, Norris K, George AL, Roden DM (2002). Allelic variants in long-QT disease genes in patients with drug-associated torsades de pointes. Circulation.

[B24] Mitcheson JS, Chen J, Lin M, Culberson C, Sanguinetti MC (2000). A structural basis for drug-induced long QT syndrome. PNAS.

[B25] Arizona CERT Drug List by Risk Groups. http://www.arizonacert.org/medical-pros/drug-lists/drug-lists.htm.

[B26] Sala M, Vicentini A, Brambilla P, Montomoli C, Jogia JRS, Caverzasi E, Bonzano A, Piccinelli M, Barale F, de Ferrari GM (2005). QT interval prolongation related to psychoactive drug treatment: a comparison of monotherapy versus polytherapy. Ann Gen Psychiatry.

[B27] WHO (2005). Collaborating Centre for Drug Statistics Methodology; ATC Index with DDDs 2006. http://www.whocc.no/atcddd/.

[B28] Al-Khatib SM, Allen LaPointe NM, Kramer JM, Califf RM (2003). What clinicians should know about the QT interval. JAMA.

[B29] Rijcken CAW, Monster TBM, Brouwers JRBJ, de Jong-van den Berg LTW (2003). Chlorpromazine equivalents versus defined daily doses: how to compare antipsychotic drug dosed?. J Clin Psychopharmacol.

[B30] Haverkamp W, Breithardt G (2005). Drug-induced sudden cardiac death. Eur Heart J.

[B31] Shah SU, Iqbal Z, White A, White S (2005). Heart and mind: psychotropic and cardiovascular therapeutics. Postgrad Med J.

